# Storage of boar semen at 17°C without conventional antibiotics in an extender containing an organic bactericidal substance

**DOI:** 10.3389/fvets.2023.1294903

**Published:** 2023-11-21

**Authors:** Anne-Marie Luther, Thu Quynh Nguyen, Jutta Verspohl, Dagmar Waberski

**Affiliations:** ^1^Unit for Reproductive Medicine/Clinic for Pigs and Small Ruminants, University of Veterinary Medicine Hannover, Hannover, Germany; ^2^Institute for Microbiology, University of Veterinary Medicine Hannover, Hannover, Germany

**Keywords:** boar semen, semen extender, bacteria, antibiotics, drug-resistance

## Abstract

**Introduction:**

Facing the global threat of antimicrobial resistance, the reduction of antibiotic use in semen extenders is a main goal in artificial insemination (AI) of pigs. The aim of this study was to investigate the potential of a commercial extender containing an organic bactericidal supplement in the absence of conventional antibiotics to control bacterial growth and to maintain the quality of boar spermatozoa during long-term semen storage for up to 144 h at 17°C.

**Methods:**

Semen from 233 boars housed at 16 European AI centers was split and diluted in the long-term extender “Androstar Plus without antibiotics + organic bactericidal supplement” (APlus) and in the control extender Beltsville Thawing Solution (BTS) with gentamicin, which is routinely used in many AI centers. Sperm motility was assessed with computer-assisted semen analysis (CASA) and membrane integrity was evaluated with flow cytometry. The number of bacteria was determined by counting colonies on agar plates.

**Results:**

At the end of storage, bacterial counts were ≥ 10^6^ CFU/mL in 10.7% of the APlus and in 0.4% of the BTS samples. At the same time, bacterial counts were only weakly correlated with sperm motility (*r* = −0.23, *p* < 0.05), and there was no correlation with sperm membrane integrity (*p* > 0.05). Among the 12 identified bacterial species in APlus samples, loss of sperm quality was exclusively observed in the presence of >10^6^ CFU/mL *Serratia marcescens* and *Klebsiella oxytoca*. Both these bacterial species, despite their known multi-drug resistance and the continuous use of gentamicin in Europe, proved sensitive to this antibiotic, thus indicating an efficient quality assurance program and responsible antibiotic use.

**Conclusion:**

Long-term storage of boar semen at 17°C without conventional antibiotics in an extender containing an organic bactericidal supplement is an option if semen samples are regularly tested for the presence of *S. marcescens* and *K. oxytoca*, and the source of contamination is eliminated.

## 1 Introduction

Antimicrobial resistance is one of the greatest global health challenges of our time. By 2030, global antimicrobial use in food-producing animals is projected to increase by 8.0% to 107.472 tonnes (1), particularly in countries with intense pork production. Following the One Health approach, the prevention of and responsible use of antibiotics in food-producing animals is a central goal (2). Considering environmental and public health concerns, sustainable breeding concepts without compromising reproductive efficiency are gaining importance. Beyond this background, prevention of multi-drug resistances in extended boar semen doses used for artificial insemination have come into focus. Loss of antimicrobial efficiency resulted in a broad use of different antibiotic classes in boar semen extenders including aminoglycosides, macrolides, polypeptides, fluoroquinolones and cephalosporins. Ideally, such conventional antibiotics must be completely eliminated from semen extenders. In this sense, legislation has recently been enforced for semen traded within the European Union (3). However, fundamentally, bacteria stemming from the male genital tract and the environment are always present in semen. Although cold storage at 5°C is a new option for antibiotic-free preservation of boar semen (4–7), semen processing and transport logistic are well established for 17°C, as this is the common storage temperature in the artificial insemination (AI) of pigs worldwide.

Semen extenders are not perfect nutrient media for bacteria, but it is obvious that bacterial counts increase at 17°C to spermicidal levels when simple first generation semen extenders like Beltsville Thawing Solution (8) without antibiotics are used (9, 10). Recent studies showed that a next generation antibiotic-free extender with intrinsic antimicrobial activity effectively inhibited the growth of commensal bacteria and preserved high sperm quality during long-term semen storage for 144 h (9). The sperm protecting effect associated with low bacterial loads was especially expressed by maintenance of high motility, which is a sensitive parameter for sperm functionality and fertilizing capacity (11). Antimicrobially active extenders offer the chance to abandon conventional antibiotics, but must be effective against a broad spectrum of seminal bacteria. This challenge applies for any potential alternative to antibiotics in semen extenders including physico-mechanical removal of microorganism, for example by Single Layer Centrifugation (12) or microfiltration (13).

Typically, a mixture of bacterial species was detected in fresh boar semen by aerobic culture, mostly Gram-negative, for example *Pseudomonas* spp.*, Proteus* spp. and *Escherichia coli*, and to a lesser extent Gram-positive, for example, *Enterococcus* spp. and *Staphylococcus* spp. (14–17). Most of them are tolerated by sperm even when present at counts of >10^6^ CFU/mL (18–21), whereas others like *Serratia marcescens* and *Klebsiella oxytoca* were frequently isolated from low-quality samples with bacteriospermia (15, 22). Seminal microbiomes differ between individual boars and AI centers (23), and consequently, antimicrobial strategies should be tested on a larger scale under different field conditions.

The aim of the present study was to examine the potential of semen storage without conventional antibiotics using a commercial long-term extender containing an organic bactericidal supplement (OBS) in semen portions processed from 233 AI boars in 16 European insemination centers. Bacterial species were determined when CFU were > 5 × 10^3^/mL to identify potential bacteria of concern, and their presence was related to sperm quality. Identification of bacteria that grow to spermicidal levels in the absence of conventional antibiotics will provide a basis for targeted developments of innovative antimicrobial concepts in pig breeding.

## 2 Materials and methods

### 2.1 Chemicals and media

Chemicals used for buffers and fluorescent dyes were of analytical grade and purchased from Sigma-Aldrich Production GmbH (Steinheim, Germany), Oxoid Deutschland GmbH (Wesel, Germany), Enzo Life Sciences GmbH (Lörrach, Germany), Carl Roth GmbH & Co. KG (Karlsruhe, Germany), Thermo Fisher Scientific, Inc. (Waltham, MA, United States), Merck KGaA (Darmstadt, Germany), and Beckman Coulter GmbH (Krefeld, Germany). The Phosphate Buffered Solution (PBS) consisted of 15 mM NaCl, 1.2 mM NaH_2_PO_4_*2H_2_O, and 0.25 mM KH_2_PO_4_. The semen extenders were obtained from Minitüb GmbH (Tiefenbach, Germany).

### 2.2 Semen samples

Semen samples were received from 233 boars located in 16 European AI centers (13 in Germany, one in Austria, one in Spain, and one in Switzerland). Semen samples used in this study were obtained exclusively from EU authorized AI centers during their routine semen dose production in accordance with the animal welfare agreement with the responsible veterinary authority. From each center, 10 to 20 semen samples were used from different boars aged between 8 and 60 months and predominately (~ 80%) from the Piétrain breed. Other breeds were Duroc, Large White, and German Landrace. Subsamples from each boar were manually extended in one-step in Beltsville Thawing solution (8) supplemented with 0.25 mg/mL gentamicin sulfate, and “Androstar Plus without antibiotics + OBS,” a long-term extender of the Androstar Plus (APlus) family lacking conventional antibiotics and supplemented with an organic bactericidal supplement (Minitüb GmbH; Ref. 13,531/7001; Minitüb (24)). The Beltsville Thawing Solution with gentamicin (BTS) was chosen as control because this is the extender routinely used in many European AI centers. Semen portions containing 20 ± 2 × 10^6^ sperm/mL in a final volume of 90 ± 10 mL were kept at room temperature for 90 to 120 min and then sent overnight in insulated boxes to the laboratory of the Unit for Reproductive Medicine, University of Veterinary Medicine Hannover, Hannover, Germany. Upon arrival, temperature in the boxes was measured with an infra-red thermometer and was between 15°C and 18°C. Semen doses were stored at 17°C in a temperature-controlled refrigerator in the dark.

### 2.3 Microbiology

Ten-fold serial dilutions in PBS ranging from10^−1^ to 10^−10^ were used to determine bacterial counts in 466 semen samples. Volumes of 100 μL were plated on Columbia agar with sheep blood (Oxoid Deutschland GmbH). After incubation for 24 to 48 h at 37°C under aerobic conditions, the bacterial colonies were counted and the total number of colony-forming units per milliliter (CFU/mL) was calculated. In semen portions with a bacterial count >5 × 10^3^ CFU/mL, bacterial species were identified with MALDI-TOF MS (microFlex LT, Bruker Daltonics GmbH & Co. KG, Bremen, Germany) with the software Biotyper (Bruker Daltonics, Server Version 4.1.100).

### 2.4 Spermatology

Sperm quality was assessed after 24 to 48 h and after 120 to 144 h of storage in 466 semen samples. Sperm motility was determined with computer-assisted semen analysis (CASA) system AndroVision® (Version 1.2, Minitüb GmbH) using 20 μL Leja chambers (Leja Products B.V., Nieuw-Vennep, the Netherlands) (5). At least 500 sperm were recorded with a frame rate of 30 pictures per 0.5 s. Motile spermatozoa were identified when their curved-line velocity was >24 μm/s and the amplitude of lateral head displacement was >1 μm. Sperm agglutination was assessed with phase contrast microscopy (Carl Zeiss Microscopy GmbH, Jena, Germany) at 200× magnification in a minimum of three different fields. The degree of agglutination was scored between 0 and 3 according to the estimated percentage of agglutinated spermatozoa as follows: 0: ≤5%, 1: >5 to 20%, 2: >20 to 40%, 3: >40%.

The integrity of sperm membranes was evaluated with the CytoFlex flow cytometer (Beckman Coulter GmbH, Krefeld, Germany) and the CytExpert 2.4. software (Beckman Coulter GmbH) using final concentrations with 1.3 μmol/L Hoechst 33342, 1.5 μmol/L propidium iodide (PI), and 2 μmol/L fluorescein conjugated peanut agglutinin (FITC-PNA) (5). Fluorescence signals were detected in 10,000 events (450/45 nm BP), FL-2 (525/40 nm BP), and FL-3 (610/20 nm BP). Spermatozoa with intact plasma membranes and acrosomes were identified by a positive Hoechst stain and negative stainings for PI and FITC-PNA, and were considered viable.

### 2.5 Statistical analysis

Data were analyzed using IBM SPSS Statistics Professional (SPSS, IBM, Inc., Armonk, NY, United States). Data were checked for normal distribution with the Shapiro–Wilk Test. For analyzing data for sperm motility and membrane integrity, a two-factorial ANOVA with repeated measurements was performed. Data were correlated with the Spearman’s rank correlation coefficient. Bacterial count data were analyzed by the Friedman Test (XLSX) with repeated measurements. To test homogeneity, a ‘χ2’-test was performed. The significance level was set at *p* < 0.05.

## 3 Results

### 3.1 Microbiology of the extended semen samples

**Figure 1:**
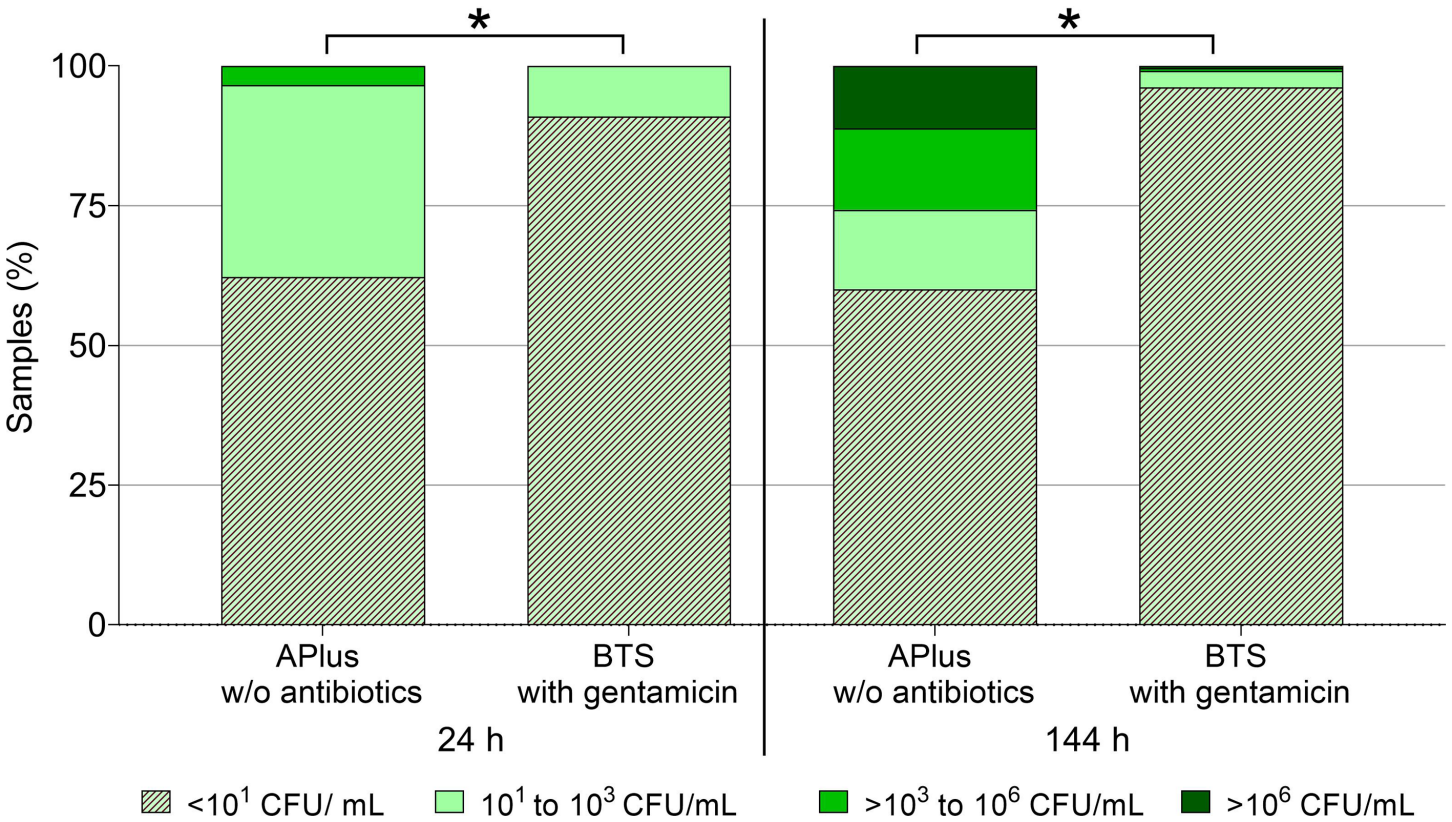
Frequency distribution of semen samples in four classes of bacterial counts (CFU/mL) at two storage time points at 17°C. Semen from 233 boars was extended as split samples in Androstar Plus without antibiotics + organic bactericidal supplement (APlus) and in Beltsville Thawing Solution (BTS) containing the antibiotic gentamicin. ^*^Values for overall sample distribution in all four CFU-classes differ between extender groups at a given time point (*p* < 0.05).

Based on the analysis of semen from 233 boars, Figure 1 shows that bacterial counts until 48 h were below the detection limit (< 10 CFU/mL) in 91.0% of samples extended in BTS with gentamicin and in 62.2% of the samples extended in the APlus extender. Bacterial counts ≥10^3^ CFU/mL were detected in none of the BTS samples and in 3.4% of the APlus samples. Up until 144 h of storage, no bacteria were detected in 96.1% of the BTS-samples and in 60.1% of the APlus samples. Bacterial counts ≥10^3^ CFU/mL were found in 0.9% of the BTS samples and in 25.8% of the APlus samples. Bacterial counts ≥10^6^ CFU/mL were detected in one (0.4%) of the BTS samples and in 11.2% of the APlus samples.

**Figure 2:**
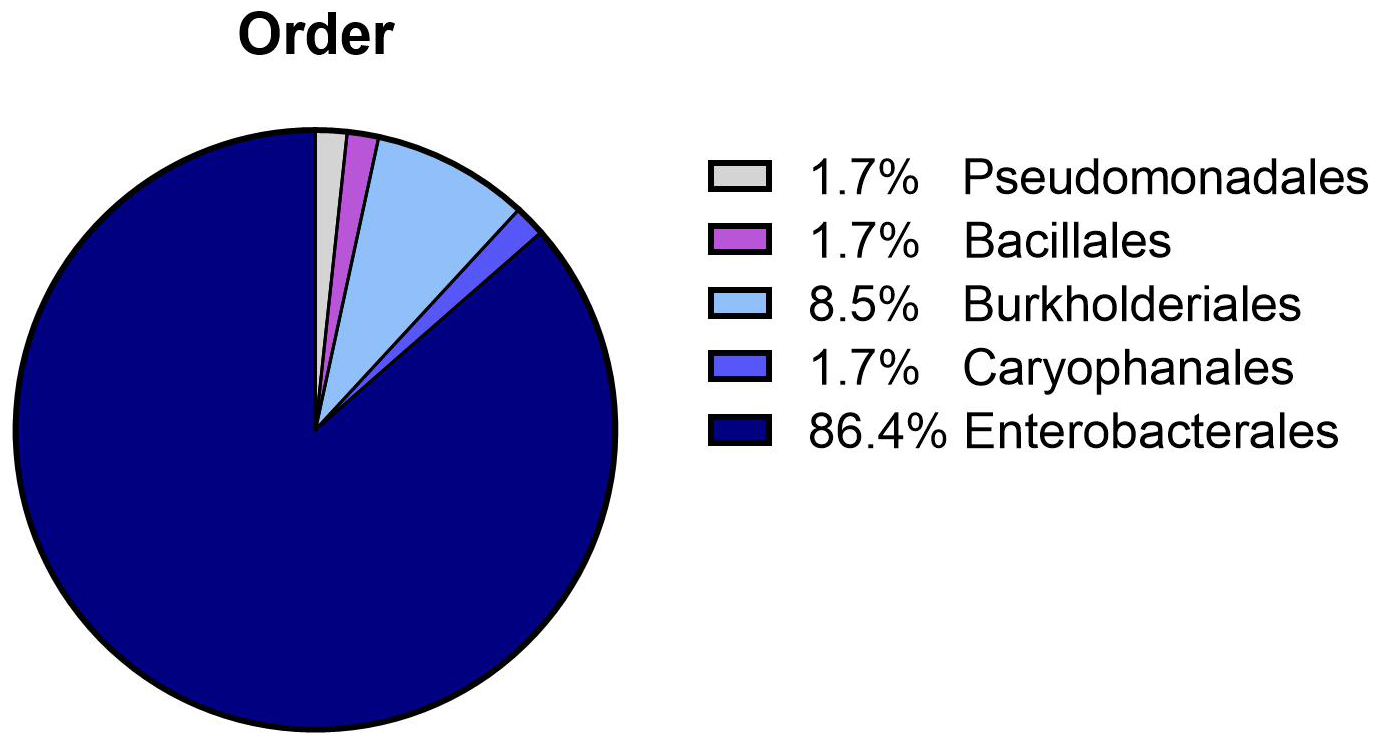
Relative abundance of bacteria orders detected in semen doses with greater than 5 × 10^3^ CFU/mL after 120 to 144 h of storage at 17°C. Bacteria above this CFU level were detected in 51 (21.9%) semen samples extended in Androstar Plus without antibiotics + organic bactericidal supplement (APlus) and in two (0.9%) semen samples extended in Beltsville Thawing Solution with the antibiotic gentamicin from 233 different boars.

Bacterial species were identified in all samples (*n* = 53; 51 in APlus and two in BTS) with >5 × 10^3^ CFU/mL. The bacteria belonged to two phyla, Protobacteria with four different orders containing 12 bacterial species, and Bacillota with the order Bacillales containing two bacterial species. The dominating order was Enterobacterales (Figure 2). The two BTS samples contained *Achromobacter xylosoxidans*. The identified bacterial species and their maximal counts in the APlus semen samples are shown in Table 1. Twelve bacterial species were Gram-negative, and two were Gram-positive (*Bacillus subtilis* and *Staphylococcus epidermidis*). *Serratia marcescens* was present at the highest abundance with bacterial counts up to 10^9^ CFU/ mL. For verifying the multi-drug resistance, exemplary susceptibility tests of two *S. marcescens* isolates from different AI centers are shown in Supplementary Table S1.

**Table 1:** Microbiology and sperm quality in semen samples extended in Androstar Plus without antibiotics + organic bactericidal supplement (APlus) containing >5 × 10^3^ CFU/mL after 120 h to 144 h of storage at 17°C.

Bacterial species	Samples	AI centers	CFU/mL		Low motility	Low membrane integrity	High agglutination
	(*n*)	(*n*)	min	max	(*n* samples)	(*n* samples)	(*n* samples)
*Achromobacter xylosoxidans*	1	1	5.6 × 10^7^	5.6 × 10^7^	0	0	0
*Alcaligenes faecalis*	1	1	8.0 × 10^5^	8.0 × 10^5^	0	0	0
*Bacillus subtilis*	1	1	2.0 × 10^7^	2.0 × 10^7^	0	0	0
*Kerstersia gyiorum*	1	1	1.5 × 10^5^	1.5 × 10^5^	0	0	0
*Klebsiella oxytoca*	3	3	2.0 × 10^5^	4.9 × 10^8^	0	0	2
*Kosakonia cowanii*	1	1	4.0 × 10^6^	4.0 × 10^6^	0	0	0
*Proteus hauseri/vulgaris/penneri*	13	7	1.0 × 10^4^	9.6 × 10^7^	0	0	0
*Proteus mirabilis*	1	1	9.0 × 10^5^	9.0 × 10^5^	0	0	0
*Providencia rettgeri*	2	1	1.5 × 10^4^	1.9 × 10^9^	0	0	0
*Providencia stuartii*	10	2	8.0 × 10^3^	1.0 × 10^6^	0	0	0
*Pseudomonas aeruginosa*	1	1	1.6 × 10^4^	1.6 × 10^4^	0	0	0
*Serratia marcescens*	19	6	1.2 × 10^4^	1.5 × 10^9^	7	4	8
*Serratia rubidaea*	2	1	9.0 × 10^3^	6.3 × 10^7^	0	0	0
*Staphylococcus epidermidis*	1	1	3.0 × 10^4^	3.0 × 10^4^	0	0	0

Sperm quality was assessed by threshold values and compared to control samples from the same boar extended in the routinely used Beltsville Thawing Solution with the antibiotic gentamicin: Low motility was defined as total motility <70%, and more than 10% immotile sperm compared to the control; low membrane integrity was defined as membrane intact sperm <85%, and more than 5% defective membranes compared to the control; high agglutination was defined as agglutination Score 3 (more than 40% sperm agglutinated) and Score < 3 in the control.

### 3.2 Microbiology and sperm quality

Among the APlus semen samples with >5 × 10^3^ CFU/mL (*n* = 57), 33.3% were positive to *S. marcescens* (Table 1). Sperm quality was reduced in eight of the 19 samples contaminated with *S. marcescens* compared to the *Serratia*-free BTS controls of the same boar. Loss of sperm quality was mainly due to high sperm agglutination and/or low sperm motility. Semen quality was also affected by high sperm agglutination in two of three samples containing *K. oxytoca*. All other bacterial species at levels >5 × 10^3^ CFU/mL up to 10^9^ CFU/mL (*Providencia rettgeri*) did not impair sperm quality compared to control samples with gentamicin.

**Figure 3:**
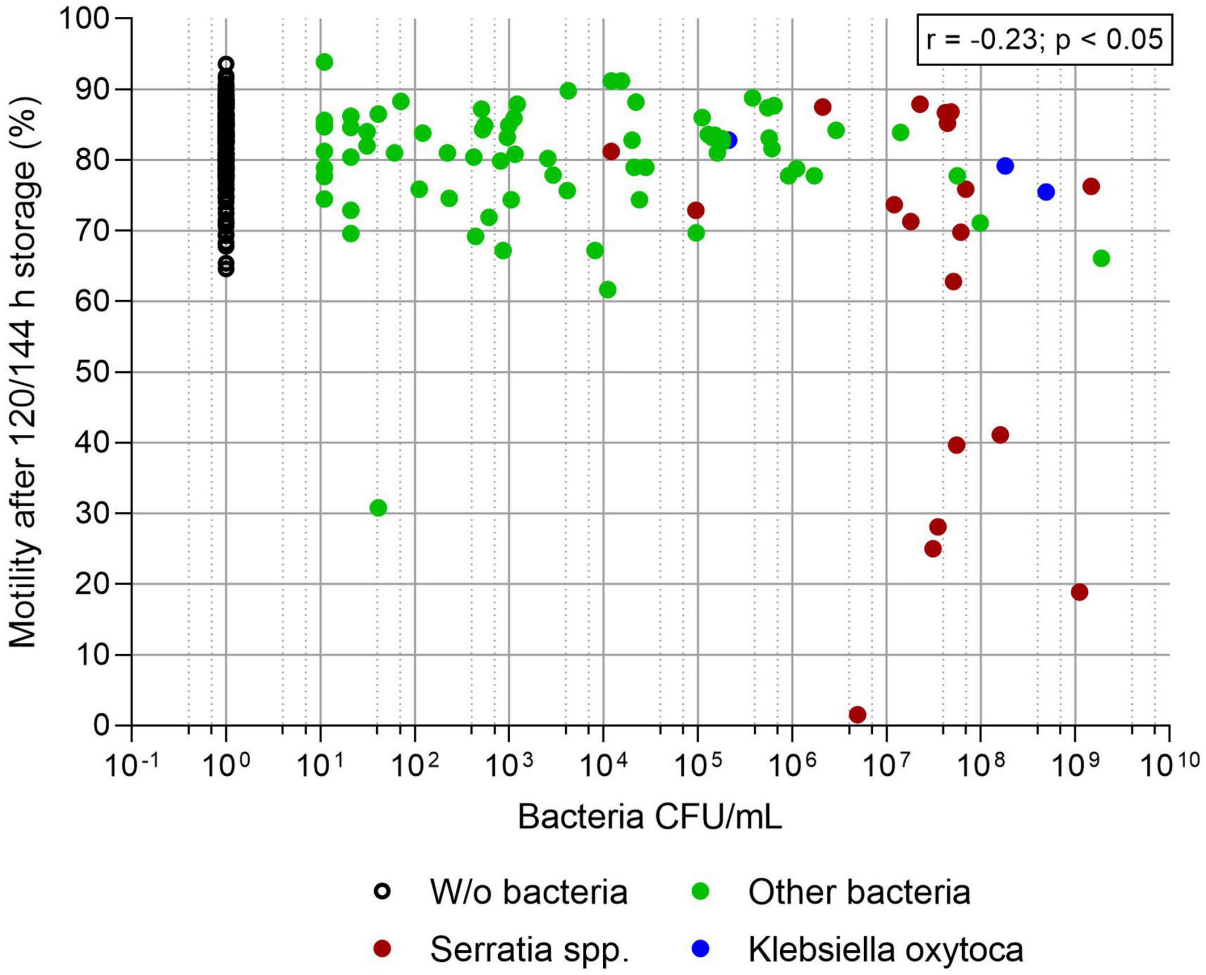
Sperm motility (%) in relation to bacterial counts (CFU/mL) in semen extended in Androstar Plus without antibiotics + organic bactericidal supplement (APlus) and stored for 120 to 144 h at 17°C; n = 233 semen samples from different boars.

**Figure 4:**
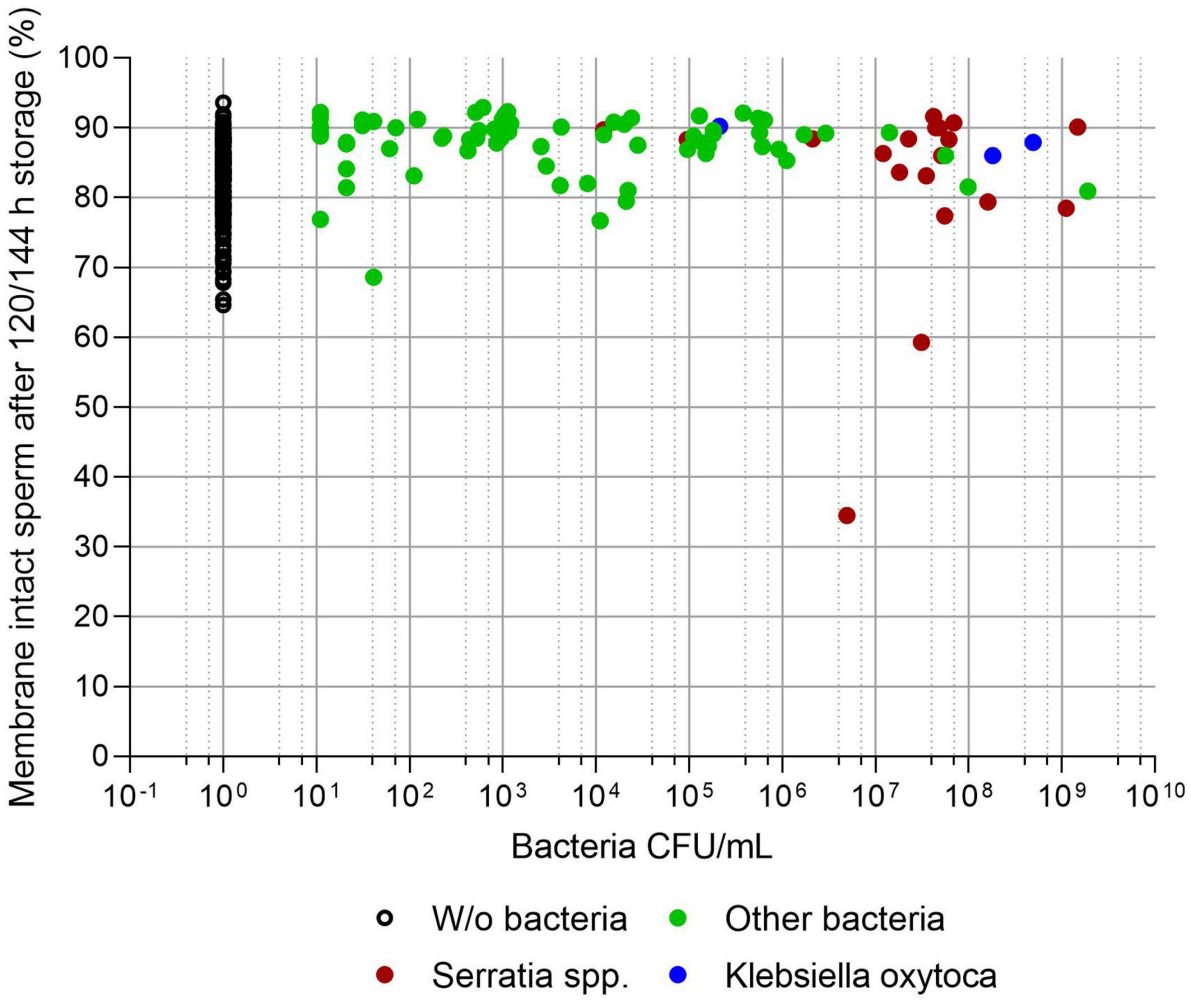
Sperm membrane integrity (% spermatozoa with intact plasma membrane and acrosome) in relation to bacterial counts (CFU/mL) in semen extended in Androstar Plus without antibiotics + organic bactericidal supplement (APlus) and stored for 120 to 144 h at 17°C; n = 233 semen samples from different boars. There was no correlation between the percentage of membrane intact spermatozoa and bacterial counts (*p* > 0.05).

Figures 3, 4 show sperm motility and membrane integrity (viability) of the 233 APlus samples after long-term storage up to 144 h in relation to their bacterial counts. In 3.0% of the samples (*n* = 10), sperm motility was less than 65% and therefore would not have been eligible for AI. Of these, all but one sample contained *S. marcescens*, whereas all three samples with *K. oxytoca* maintained high sperm motility and membrane integrity but showed an increased degree of agglutination (see Table 1). Sperm membrane integrity was affected in three samples, two of them with *S. marcescens*.

**Figure 5:**
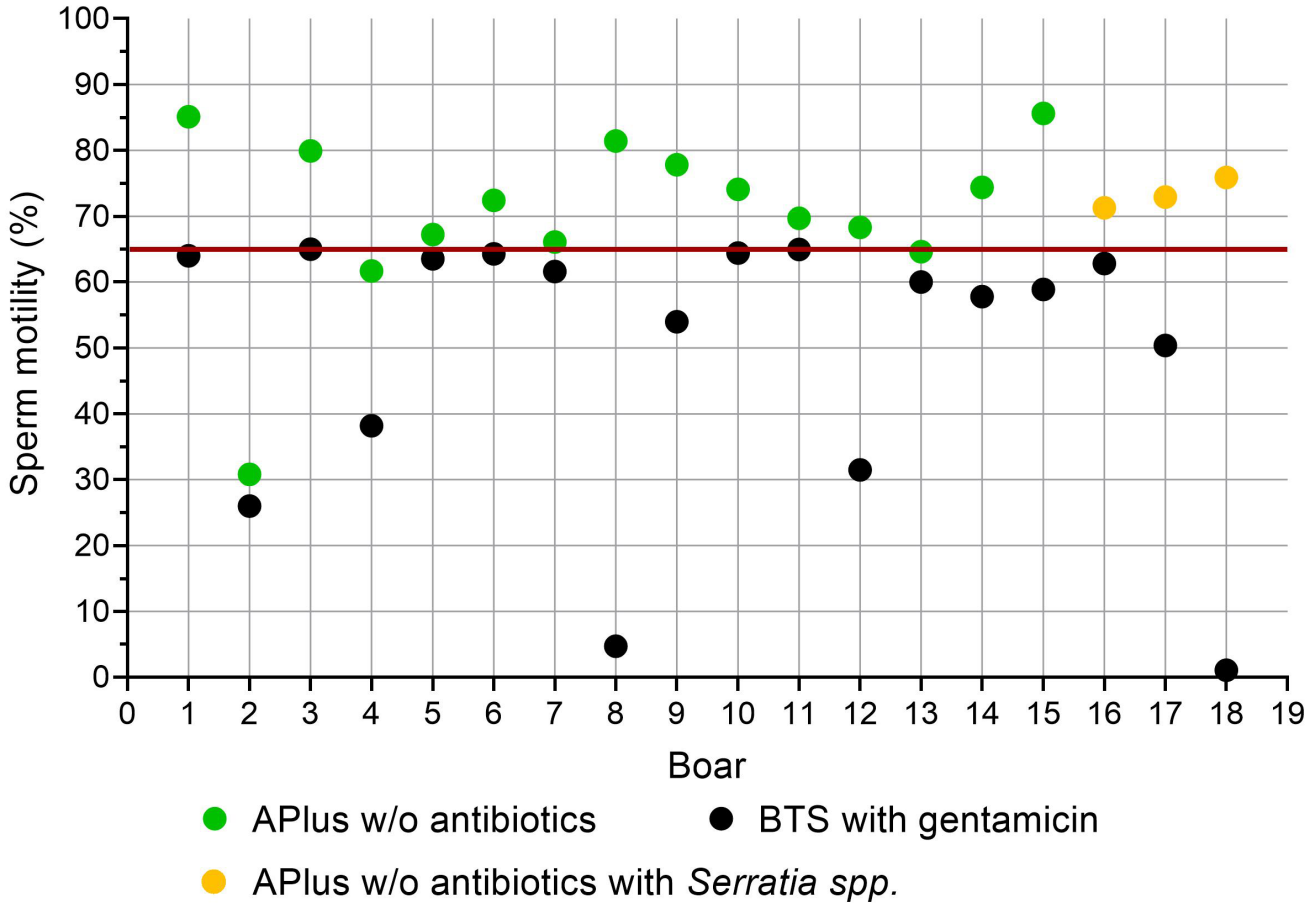
Sperm motility (%) in low-quality semen samples extended in Beltsville Thawing Solution (BTS) with the antibiotic gentamicin (n = 18, 7.7% from 233 boars) compared to semen samples from the same boar extended in Androstar Plus without antibiotics + organic bactericidal supplement (APlus). Samples were analyzed after storage for 120 to 144 h at 17°C.

Figure 5 shows sperm motility in low quality BTS-samples compared to APlus of the same boar. Eighteen samples stored in BTS with gentamicin up to 144 h showed motility below the thresholds for useable semen (< 65%; (25)). Fifteen samples from the same ejaculates extended in APlus maintained motility above the threshold value despite the presence of moderate counts (up to 6.9 × 10^7^ CFU/mL) of *S. marcescens* in three of the samples.

## 4 Discussion

The present study shows that long-term semen preservation in the absence of conventional antibiotics at 17°C in an extender of the Androstar Plus family containing OBS maintained high sperm quality in 97.7% of the samples collected from 233 boars in 16 AI centers, even in the presence of bacteria loads up to log levels of 10^9^ CFU/mL. Loss of bacteria-induced sperm quality was exclusively associated with the presence of *S. marcescens* and *K. oxytoca* at >10^6^ CFU/mL. This threshold level is in agreement with previous reports on the long-term exposure of boar sperm to these bacterial species (26), and other opportunistic pathogen bacterial species typically detected in boar semen, for example *E. coli, Enterobacter cloacae, Proteus vulgaris* and *Pseudomonas aeruginosa* (18–21, 27, 28). The results strengthen the notion that *S. marcescens* and *K. oxytoca* should be the main target of any antimicrobial concept for the liquid preservation of boar semen.

All other 12 bacterial species identified here in the absence of conventional antibiotics did not impair sperm motility or viability even when present at >10^6^ CFU/mL. In contrast, sperm motility was maintained at a high level despite the presence of bacteria compared to antibiotic-containing BTS-samples with poor motility. This does not seem surprising given the fact that BTS is a short-term extender, and it also shows that the long-term storage ability of semen extenders is more important for preserving sperm shelf life than the complete eradication of bacteria.

Compared to short-term semen extenders, long-term preservation media usually provide higher sperm protection against different stressors like temperature fluctuation (29, 30) or long-term exposure of seminal plasma during semen storage (31). Furthermore, they may also protect sperm better from bacterial stress, for example, caused by bacterial secretions (32) or binding to glycoprotein receptors on the sperm surface (33). Alternatively or additionally, long-term extenders without conventional antibiotics may have intrinsic antimicrobial effects which reside in the interaction of extender ingredients (9) or the activity of specific antimicrobial supplements such as antimicrobial peptides, nanoparticles phytoextracts, or other natural components (reviewed by Schulze et al. (34), Contreras et al. (35)). The underlying antimicrobial effective mechanisms in commercial extenders remain unexplored because their composition is not published. In any case, extender developments and performance testings for long-term sperm preservation should also include their intrinsic antimicrobial activity as a basis to reduce or replace conventional antibiotics in porcine AI.

A surprising finding of our study was that in six AI centers *S. marcescens* and *K. oxytoca* were not detected in any of the control samples extended in BTS with gentamicin. The high sensitivity of this classic antibiotic to these bacteria and also other bacterial species was unexpected because it has been used continuously in European AI centers for decades (36), and loss of activity has been repeatedly reported (10, 37, 38). It is to note that 15 of the 16 AI centers involved in the present study regularly participate in a science-based Quality Assurance (QA) program led by the two spermatology reference laboratories of the German Livestock Association (BRS). This indicates a high efficiency of the multitude of preventive QA strategies against the development of drug-resistance, that include systematic hygiene management with regular checks of critical control points, the prudent use of antibiotics, continuous training of the laboratory personnel, and regular semen quality monitoring by third party reference laboratories (36, 39, 40). Although gentamicin was still efficient, it cannot be ignored that the presence of *S. marcescens* and *K. oxytoca* in AI centers is a cause for concern. Being known as typically multi-drug-resistant microbes (41) with fast growth rates in extended semen (22, 26), they will, sooner or later, develop resistance against gentamicin or any other still efficient antibiotics (42). Ideally, testing of semen samples in extenders lacking conventional antibiotics should be implemented in the QA program for early detection of those bacterial species before resistance is acquired. Noteworthy, antibiotic-free semen storage in a cold-shock protective extender provides an efficient tool to prevent the growth of bacteria, including *S. marcescens* and *K. oxytoca* (5).

During natural mating, large quantities of the genital bacteria are transmitted to the female reproductive tract, particularly in the porcine species where up to 500 mL of semen are slowly infused for 10 min or longer into the uterus. Co-evolution of bacteria and higher organisms (43) and studies on the interaction between the seminal and the female microbiome suggest that commensal bacteria originating from the male genital tract could even enhance reproductive efficiency (44). The bacterial spectrum identified here in long-term stored semen in the absence of conventional antibiotics resembles the relative abundance of phyla described in recent microbiome studies of boar semen using RNA (23, 45) and DNA sequencing (46). This indicates that the semen preservation without conventional antibiotics maintains bacterial diversity, whereas the use of the classic antibiotic gentamicin induces a shift (10) and decrease (4) in the relative abundance of bacterial species, which might compromise the suggested positive effects on fertility.

## 5 Conclusion

Long-term storage at 17°C without conventional antibiotics using an extender with intrinsic antimicrobial activity is an option if AI centers apply rigorous hygiene practices in conjunction with regular screening for the early detection and elimination of critical bacteria such as *S. marcescens* and *K. oxytoca*. Acceptance of sub-spermicidal bacterial counts by breeding organizations is encouraged to prevent the overuse of conventional antibiotics and the associated development of drug resistance. Testing of semen extenders for long-term semen preservation ideally would consider antimicrobial efficiency in the absence of conventional antibiotics, with a special focus on drug-resistant bacteria.

## Data availability statement

The raw data supporting the conclusions of this article will be made available by the authors, without undue reservation.

## Ethics statement

Ethical approval was not required for the study involving animals in accordance with the local legislation and institutional requirements because Semen samples were obtained from EU-authorized AI centers as subsamples from ejaculates collected for their routine production of semen doses. All AI centers are obliged to animal welfare rules and are under supervision of their local veterinary authority. There were no interventions according to the Animals Welfare Act.

## Author contributions

A-ML: Conceptualization, Data curation, Formal analysis, Investigation, Methodology, Validation, Visualization, Writing – original draft. TN: Investigation, Writing – review & editing. JV: Supervision, Writing – review & editing. DW: Conceptualization, Funding acquisition, Methodology, Project administration, Resources, Supervision, Validation, Writing – review & editing.
