# Supplementary material for: Storage of boar semen at 17°C without conventional antibiotics in an extender containing an organic bactericidal substance
Source: Front Vet Sci. 2023 Nov 21;10:1294903. doi: 10.3389/fvets.2023.1294903 (PMC10699307; doi:10.3389/fvets.2023.1294903)
Supplement: Supplementary file 1 [file Table_1.DOCX]

Supplementary Material:

**Table S1:** Susceptibility test (MIC) of two bacterial species isolated from semen extended in Androstar^®^ Plus extender without antibiotics. The semen samples originated from two different artificial insemination centers.

|  | Bacterial species | |
| --- | --- | --- |
| Antibiotic | *Serratia marcescens*  F/22/002064/M | *Serratia marcescens*  F/22/005570/M |
| Amoxicillin /  Clavulanic acid | >= 32/16 | >= 32/16 |
| Ampicillin | >= 32 | >= 32 |
| Ceftiofur | 2 | 0.5 |
| Cephalothin | >= 32 | - |
| Colistin | >= 4 | >= 4 |
| Enrofloxacin | 0.063 | 0.125 |
| Erythromycin | >= 8 | >= 8 |
| Florfenicol | >= 16 | >= 16 |
| Gentamicin | 0.5 | 0.5 |
| Penicillin G | >= 16 | >= 4 |
| Spectinomycin | 64 | - |
| Tetracyclin | >= 16 | >= 16 |
| Tiamulin | >= 64 | >= 32 |
| Tilmicosin | >= 32 | >= 32 |
| Trimethoprim / Sulfonamid | <= 0.25/4.8 | <= 0.25/4.8 |
| Tulathromycin | >= 128 | >= 128 |

Resistance testing is performed by microdilution method according to CLSI Vet01, CLSI Vet01S, CLSI Vet06, CLSI M100S or According to scientifically proven procedures.

The numerical value corresponds to the Minimum Inhibitory Concentration (MIC) of the active substance in µg/ml.
